# Antineoplastic effects of an Aurora B kinase inhibitor in breast cancer

**DOI:** 10.1186/1476-4598-9-42

**Published:** 2010-02-22

**Authors:** Christopher P Gully, Fanmao Zhang, Jian Chen, James A Yeung, Guermarie Velazquez-Torres, Edward Wang, Sai-Ching Jim Yeung, Mong-Hong Lee

**Affiliations:** 1 Department of Molecular and Cellular Oncology, The University of Texas MD Anderson Cancer Center, Houston, TX 77030, USA; 2 The University of Texas Graduate School of Biomedical Sciences at Houston, Houston, TX 77030, USA; 3 The University of Texas MD Anderson Cancer Center, Program in Genes and Development, Houston, TX 77330, USA; 4 Department of General Internal Medicine, Ambulatory Treatment & Emergency Care, The University of Texas MD Anderson Cancer Center, Houston, TX 77030, USA; 5 Department of Endocrine Neoplasia and Hormonal Disorders, The University of Texas MD Anderson Cancer Center, Houston, TX 77030, USA; 6 The University of Texas MD Anderson Cancer Center, Program in Cancer Biology, Houston, TX 77330, USA

## Abstract

**Background:**

Aurora B kinase is an important mitotic kinase involved in chromosome segregation and cytokinesis. It is overexpressed in many cancers and thus may be an important molecular target for chemotherapy. AZD1152 is the prodrug for AZD1152-HQPA, which is a selective inhibitor of Aurora B kinase activity. Preclinical antineoplastic activity of AZD1152 against acute myelogenous leukemia, multiple myeloma and colorectal cancer has been reported. However, this compound has not been evaluated in breast cancer, the second leading cause of cancer deaths among women.

**Results:**

The antineoplastic activity of AZD1152-HQPA in six human breast cancer cell lines, three of which overexpress HER2, is demonstrated. AZD1152-HQPA specifically inhibited Aurora B kinase activity in breast cancer cells, thereby causing mitotic catastrophe, polyploidy and apoptosis, which in turn led to apoptotic death. AZD1152 administration efficiently suppressed the tumor growth in a breast cancer cell xenograft model. In addition, AZD1152 also inhibited pulmonary metastatic nodule formation in a metastatic breast cancer model. Notably, it was also found that the protein level of Aurora B kinase declined after inhibition of Aurora B kinase activity by AZD1152-HQPA in a time- and dose-dependent manner. Investigation of the underlying mechanism suggested that AZD1152-HQPA accelerated protein turnover of Aurora B via enhancing its ubiquitination.

**Conclusions:**

It was shown that AZD1152 is an effective antineoplastic agent for breast cancer, and our results define a novel mechanism for posttranscriptional regulation of Aurora B after AZD1152 treatment and provide insight into dosing regimen design for this kinase inhibitor in metastatic breast cancer treatment.

## Background

Aurora kinases are a family of serine/threonine kinases which share ~70% homology in their kinase domains [1-3], and they are essential in cell cycle control and mitosis [3-6]. Mammalian cells have three Aurora family members: A, B and C. The Aurora kinases are expressed at maximum levels during mitosis. Aurora A and B have different subcellular localizations and serve distinct functions during mitosis. Together, they tightly manage chromosome segregation to ensure that each resulting daughter cell receives a full complement of chromosomes [3-5]. All three Aurora kinases have been shown to be amplified or overexpressed in human cancer and contribute to tumorigenesis through their link to invasive disease and genomic instability [3]. By virtue of their important role in cell proliferation and their oncogenic potential [7], the Aurora kinases are potentially important targets for cancer therapeutics.

In contrast to Aurora A which localizes to the centrosomes and contributes to spindle bi-polarity by managing microtubule assembly and centrosome organization, Aurora B maintains correct kinetochore-microtubule attachments and is localized to the chromosomes during metaphase. Aurora B relocalizes to the midbody of the cell during late anaphase and telophase, which hints at an additional function during cytokinesis [4]. Aurora B phosphorylates Histone H3 at serine 10 leading to dissociation of Histone H1 and chromatin condensation [8]. As a regulator of chromosome segregation, Aurora B is part of the Chromosome Passenger Complex (CPC) which includes its substrates: INCENP, Borealin and Survivin. The CPC governs the spindle checkpoint and manages correct microtubule attachments to kinetochores [6-9].

Aurora B has been demonstrated to be overexpressed in multiple myeloma [10], AML [1], colorectal [7], prostate [11] and pancreatic [12] cancers. In human breast cancer, an oncogenic link to Aurora B has not been made although Aurora A may be overexpressed in 95% of cases [13] and may be used as a predictor of survival [14]. Overexpression of Aurora A may not simply be a gain of oncogenic function, rather Aurora A may be interfering with the delicate balance of Aurora B in the cell [15]. Aurora A kinase activating mutations do not further enhance the transformation phenotype of Aurora A [16]. Inhibition kinase activities of both Aurora A and B by ZM447439, a pan Aurora kinase inhibitor, results in cellular changes that most resemble loss of Aurora B function [15], and mutations in Aurora B confer resistance of HCT116 cells to ZM447439 [17]. Therefore, Aurora B may in fact be a more important drug target than Aurora A.

In this study, the knowledge gap regarding the use of the Aurora B-specific inhibitor, AZD1152, in breast cancer is addressed. AZD1152 is a dihydrogen phosphate prodrug and is metabolized in the serum to its active form, AZD1152-HQPA [hydroxyquinazoline pyrazol anilide], which is a small molecule ATP binding pocket competitor [18]. AZD1152-HQPA has potent selectivity for inhibition of Aurora B [K_i _= 0.36 nM] compared with Aurora A [K_i _= 1,369 nM] and a panel of 50 other kinases [7]. The antineoplastic effect of this drug has been demonstrated in human cancer cell lines, including colon, lung, and cervix [7], as well as leukemia cell lines and primary acute myeloid leukemia cultures [1].

Also evaluated were the dose-responses of AZD1152-HQPA in 6 human breast cancer cell lines as well as the cellular consequences of Aurora B inhibition. Further, the antineoplastic effects of AZD1152 in nude xenograft mice using two breast cancer cell lines were examined. This was followed by the novel discovery that this Aurora B kinase inhibitor downregulated Aurora B protein level by increasing polyubiquitination and proteasomal degradation of Aurora B. Together, these studies indicate that AZD1152 has antineoplastic activity in human breast cancer cells and that AZD1152's impact on Aurora B protein stability is another important layer of regulation that has not been characterized before.

## Results

### Breast cancer cells are sensitive to AZD1152-HQPA in vitro and display signs of mitotic catastrophe after exposure

**Figure 1:**
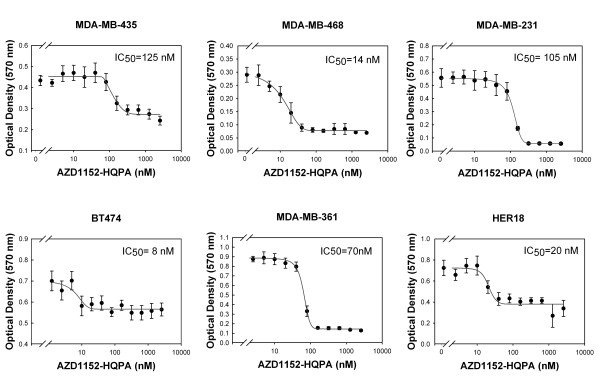
**AZD1152-HQPA inhibits breast cancer cell proliferation**. Live cells were measured by MTT assay. Log(dose)-response curves for HER18, MDA-MB-468, MDA-MB-435, MDA-MB-231, MDA-MB-361 and BT474 are shown as labeled. Each data point represents the mean of at least 4 replicates; error bars represent 95% confidence intervals. Each IC_50 _was calculated based on sigmoidal curve fitting to the respective data set.

To evaluate the effect of AZD1152-HQPA on breast cancer cells, HER18 (ER+, PR+, p53wt) breast cancer cells, which stably overexpress HER2 (parent line, MCF-7) [19], were treated with AZD1152-HQPA. Cell proliferation was measured by MTT assay (Figure 1). The concentration that achieves 50% of maximal inhibition of proliferation (IC_50_) of the cells was measured at 20 nM by sigmoidal curve fitting. Similar results were obtained in MDA-MB-468 (HER2+, EGFR+++, Pten-, ER-, PR-, p53 mutant), MDA-MB-435 (HER2+[16], ER-, PR-, p53 mutant), MDA-MB-231 (HER2+[16], ER-, PR-, p53 mutant), MDA-MB-361 (HER2+++, ER+, PR+, p53wt) and BT-474 (HER2+++[14], ER+, PR+, p53 mutant) at IC_50 _of 14 nM, 125 nM, 105 nM, 70 nM and 8 nM respectively. All human breast cancer cell lines tested showed sensitivity to AZD1152-HQPA with typical sigmoidal log (dose)-response curves. The observed IC_50_s are comparable to those found in leukemia and other human cancer cell lines [1,7]. To verify the response to drug inhibition by MTT assay, a separate dose-response assay in HER18 cells measuring proliferation inhibition by live cell count, which corroborated the IC_50 _of 20 nM for HER18 cells (data not shown), was performed. These results suggest that AZD1152-HQPA is an effective inhibitor of human breast cancer cells by MTT assay, a measure of both cell proliferation and cell death. Further, this effect was seen in various cell lines with multiple molecular profiles for HER2, ER, PR and p53.

### Treatment with AZD1152 causes mitotic catastrophe, G2/M arrest and polyploidy/aneuploidy

**Figure 2:**
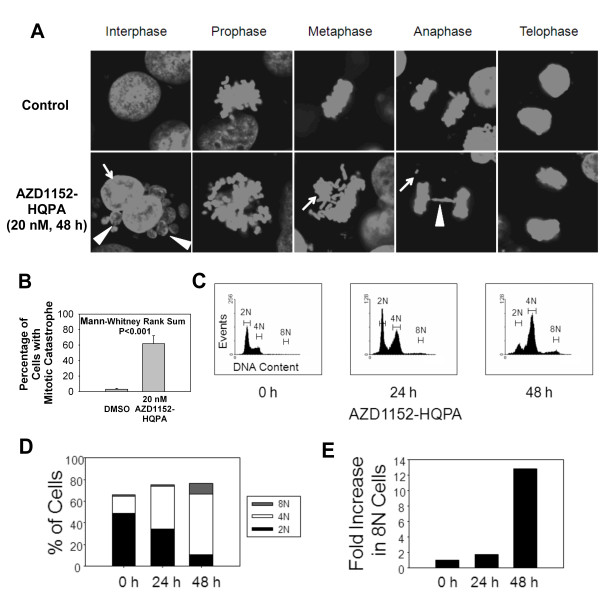
**AZD1152-HQPA causes mitotic defects, aneuploidy and polyploidy in breast cancer cells**. (A) HER18 cells were treated with control culture medium or 20 nM AZD1152-HQPA for 48 hours, stained with DAPI and examined using fluorescence microscopy. Interphase cells showed bi-nucleation (arrow) and micronuclei (arrow heads). Metaphase cells showed misaligned chromosomes (arrow). Anaphase cells showed misegregated chromosomes (arrow) and chromosomal bridges (arrow head). (B) Percentage of cells from A with mitotic catastrophe is plotted for vehicle and 20 nM AZD1152-HQPA treatments. (C) HER18 cells were treated with AZD1152-HQPA (100 nM) for indicated times and stained with propidium iodide prior to analysis of DNA content by flow cytometry. Gating indicates 2N (G1), 4N (G2/M) and 8N (polyploid) cells. Aneuploid cells were present between 4N and 8N after 48 h treatment. (D) Percentages of 2N, 4N and 8N cells are plotted at 0, 24 and 48 hour time points based on the data in C. (E) Number of 8N cells relative to 0 hours.

Given the important role of Aurora B kinase in mitosis, the impact of AZD1152-HQPA on chromosome segregation was investigated. HER18 cells were incubated with or without 20 nM AZD1152-HQPA for 48 hours. Chromosomal DNA was stained with DAPI and mitotic cells were observed by fluorescence microscopy. While control HER18 cells showed normal morphology (Figure 2A), cells treated with AZD1152-HQPA demonstrated characteristics of mitotic catastrophe including multi-nucleation, micronuclei and chromosome bridges (arrows and arrow heads). Quantification of this effect was performed and it was found that approximately 60% of drug-treated cells exhibited signs of mitotic catastrophe versus 3% for control cells (Figure 2B). These observations are consistent with the effects of loss of Aurora B function and demonstrate that AZD1152-HQPA is effective in causing mitotic catastrophe in breast cancer cells.

Measurement of the DNA content of individual HER18 cells treated with AZD1152-HQPA using flow cytometry after propidium iodide (PI) staining of DNA revealed that the percentage of 4N HER18 cells increased after treatment with AZD1152-HQPA 20 nM (Figure 2C). Importantly, HER18 cells with DNA content greater than 4N began to appear after 48 hours of exposure to AZD1152-HQPA. The percentages of 2N, 4N and 8N cells were quantified from the flow cytometry data and are displayed in Figure 2D. It can be seen that as G2/M (4N) cell percentages increase, there is a concomitant decrease in G1 (2N) cell percentages. Polyploid (8N) cells increased from 1.35% of the cell population at 24 hours to 9.7% at 48 hours. In Figure 2E, polyploid cell numbers were analyzed relative to the number of 8N cells at time 0. By 48 hours, the number of 8N cells had increased 12.8 fold. This evidence, together with the observation of nuclear morphologic changes reported above, indicates that cells become polyploid or aneuploid as mitotic catastrophe occurs after treatment with AZD1152-HQPA.

### AZD1152-HQPA causes apoptosis and decreases clonogenic potential in breast cancer cells

**Figure 3:**
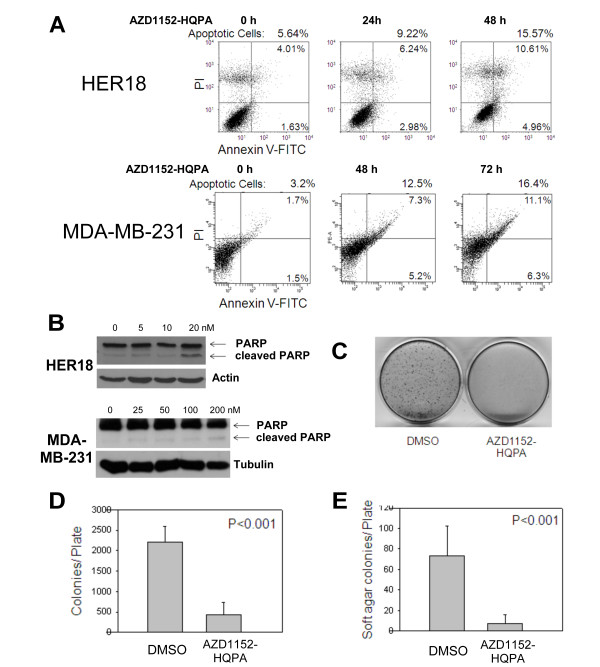
**AZD1152-HQPA induces apoptosis and reduces clonogenic potential in breast cancer cells**. (A) HER18 and MDA-MB-231 cells were exposed to AZD1152-HQPA (100 nM and 105 nM) for up to 72 hours and cells were stained with Annexin V-FITC plus propidium iodide. Samples were analyzed by flow cytometry for PI (y-axis) and Annexin V (x-axis) at 3 time points. Representative scattergrams are shown with percentages of cells displayed for each quadrant: Lower Left - live cells, Lower Right - early apoptosis, Upper Right - late apoptosis and Upper Left - necrotic cells. Overall percent apoptosis was calculated for each time point by adding Lower Right and Upper Right quadrants. (B) Apoptosis of HER18 and MDA-MB-231 cells was analyzed by Western blotting with anti-PARP antibody. PARP appears at 115 KD while the apoptotic indicator, cleaved PARP, appears at 85 KD. (C) HER18 cells were plated in triplicate at 5000 cells/plate in 6 cm tissue culture dishes and treated with either vehicle or 40 nM AZD1152-HQPA. Representative plates are shown after incubation for 12 days and staining with crystal violet. (D) Mean colony numbers from C are plotted for AZD1152-HQPA versus control. (E) Soft agar clonogenic assay was performed using HER18 cells. Mean soft agar colony numbers from triplicate plates are plotted for AZD1152-HQPA versus control. All Error bars represent 95% confidence intervals.

Next, AZD1152-HQPA treatment as the cause of cell death via apoptosis was investigated. HER18 cells were treated with AZD1152-HQPA (100 nM) for up to 48 hours, followed by staining with Annexin V-FITC/PI and analysis by flow cytometry (Figure 3A, upper panels). Percentages of cells in both early (lower right panel) and late (upper right panel) apoptosis increased with increasing duration of exposure to AZD1152-HQPA. After 24 hours of treatment, there were 9.22% apoptotic cells and 15.57% at 48 hours, compared to 5.64% in the untreated control cells (Figure 3A). Similar results were obtained with the parent cell line MCF7 (data not shown) as well as MDA-MB-231 (Figure 3A, lower panels). Apoptosis following AZD1152-HQPA treatment was also examined by immunoblotting to detect specific PARP (Poly [ADP-Ribose] Polymerase) cleavage by Caspase-3 in HER18 and MDA-MB-231 cells (Figure 3B). Increases in cleaved PARP in both cell types treated with AZD1152-HQPA were observed. This corresponds with the IC_50 _for these cell lines (Figure 1) and these results confirm that treatment with AZD1152-HQPA leads to apoptosis.

To determine if AZD1152-HQPA can inhibit the colony forming potential of breast cancer cells, HER18 cells were incubated at a low density with either control culture medium or 40 nM AZD1152-HQPA. After 12 days, colonies were stained with crystal violet and counted (Figure 3C). AZD1152-HQPA inhibited the ability of HER18 cells to form colonies (Figure 3C and 3D). The difference in the mean colony numbers per cm^2 ^between the control and AZD1152-HQPA groups was significant (P < 0.001, two-sided Student's t-test). Next, the ability of AZD1152-HQPA to inhibit anchorage independent growth of breast cancer cells was examined by using the soft agar colony forming assay. The mean colony number in soft agar was also significantly (P < 0.001, two-sided Student's t-test) decreased by 80 nM AZD1152-HQPA compared with the control in both (Figure 3E).

Taken together, these results indicate that AZD1152-HQPA has the ability to inhibit breast cancer cells by inducing apoptosis and decreasing their colony forming potential in both anchorage dependent and independent environments.

### Aurora B inhibition by AZD1152 inhibits tumor growth in an orthotopic xenograft nude mouse model

**Figure 4:**
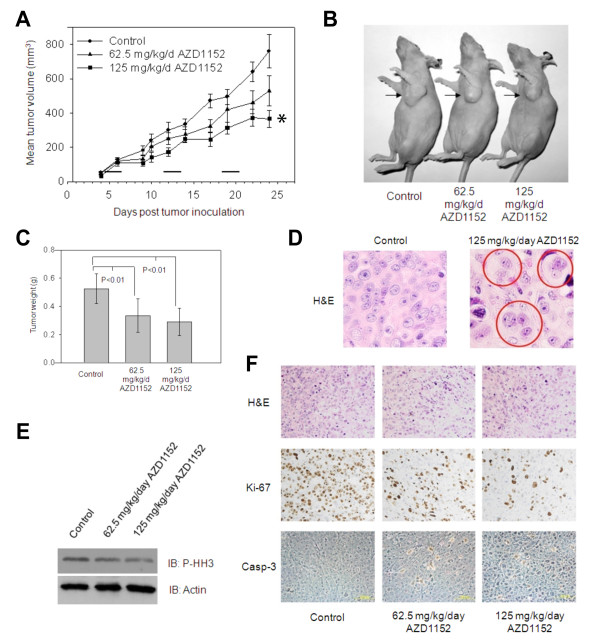
**AZD1152 inhibits orthotopically xenografted HER18 cells in nude mice**. (A) Tumor growth curves for orthotopically implanted HER18 cells are shown. Control mice received vehicle (0.3 M Tris pH 9.0), low dose mice received 62.5 mg/kg/dose AZD1152, and high dose mice received 125 mg/kg/dose AZD1152. Treatment began when tumors were measurable (approximately 50 mm^3^). Injections were given IP on days 1 and 2 of 7-day cycles, indicated by horizontal bars just above the x-axis. * P < 0.001 calculated as a mixed linear model. Error bars represent SE. (B) Representative mice bearing xenografts (arrows) from each treatment group. (C) Weights of dissected xenografts are plotted as means for each treatment group. Error bars represent 95% confidence intervals. P values were determined by one-way ANOVA. (D) H&E stained control and high dose (125 mg/kg/day) tumor samples are shown. Multi-nucleate cells (circles) were observed in tumor sections from AZD1152-treated mice but not in control mice. (E) Western blots using anti-phospho-Histone H3 and anti-actin are shown as labeled. (F) Histology and immunohistochemistry of xenografts are shown: top panels- H&E staining; middle panels- immunohistochemistry with antibodies for Ki-67; lower panels- cleaved Caspase 3.

To evaluate the *in vivo *effect of AZD1152 against aggressive breast cancer, HER18 human breast cancer cells were xenografted orthotopically into nude mice. 8.5 × 10^6 ^HER18 cells were injected into the mammary fat pad of each female athymic *nu/nu *mice that received weekly subcutaneous estradiol cypionate injections (3 mg/kg/dose) [20,21] starting 2 weeks prior to injection of tumor cells. When tumors were measurable (approximately 50 mm^3^, see Figure 4A), the mice were randomized into 3 groups: control (n = 6) which received 200 μl of 0.3 M Tris pH 9.0, low dose (n = 8) which received 62.5 mg/kg/dose AZD1152, and high dose (n = 6) which received 125 mg/kg/dose AZD1152. Doses of AZD1152 were based on the pharmacokinetic findings of previous publications that a dose of 10-150 mg/kg/day produced sufficient plasma concentrations of AZD1152 in nude mice [7,18]. Injections were administered IP on days 1 and 2 of a 7-day repeating cycle for 3 cycles. The mice treated with high dose AZD1152 showed reduced tumor volume compared to control mice (P < 0.001) and low dose treated mice showed a near-significant reduction (P < 0.08) (Figure 4A and 4B). Excised tumors in the drug-treated groups weighed significantly (P < 0.01, one-way ANOVA, post-hoc intergroup comparison using Tukey test) less than those in the control mice (Figure 4C).

Formalin-fixed tumor samples were embedded in paraffin, and sections were examined by microscopy. Consistent with the *in vitro *data from Figure 2, multinucleate cells were observed on the hematoxylin- and eosin-stained (H&E) histological slides of tumor samples from AZD1152-treated mice but not on the slides of tumors from control mice (Figure 4D). Tumor samples were also snap-frozen in liquid nitrogen, and proteins were subsequently extracted for immunoblotting. Aurora B is known to phosphorylate serine 10 of Histone H3 [22] to aid in chromatin condensation by dissociating Histone H1 from heterochromatin [23,24]. Therefore, inhibition of Aurora B kinase activity can be verified by immunoblotting with phospho-specific antibody to serine 10 of Histone H3 [8]. A reduction in phospho-Histone H3 in AZD1152-treated tumors compared with control tumors (Figure 4E) was found, confirming that AZD1152 inhibited Aurora B kinase activity *in vivo *(i.e., confirming the pharmacodynamic mechanism). Immunohistochemistry staining for Ki-67 (a cell proliferation marker) and cleaved Caspase 3 (a marker of apoptosis) revealed that Ki-67 was markedly reduced in both drug treated groups when compared with the control group and that Caspase 3 cleavage was increased in the drug-treated groups versus the control. This provides evidence that AZD1152 induced apoptosis in breast cancer cells and inhibited breast cancer cell proliferation *in vivo *(Figure 4F).

### AZD1152 inhibits breast cancer metastasis

**Figure 5:**
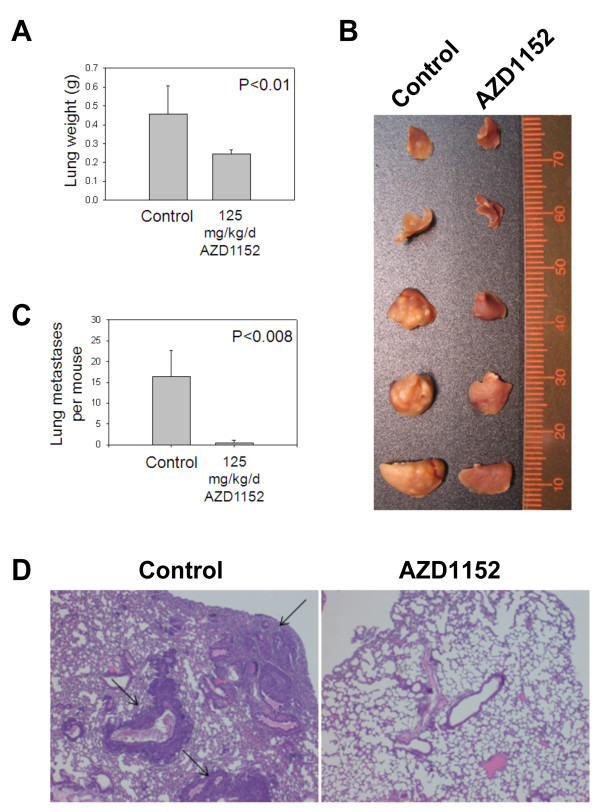
**AZD1152 inhibits growth of lung tumor nodules in a lung metastasis nude mouse model**. (A) Two million MDA-MB-231 human breast cancer cells were injected via tail veins of nude mice. AZD1152 (125 mg/kg/dose) or vehicle were administered IP on days 1 and 2 of 7-day repeating cycle for 4 cycles starting 2 days after cancer cell injection. Ten weeks after cancer cell injection, the mice were sacrificed and lungs were weighed and plotted as the means for each treatment group. (B) Matched lobes of the lungs from a representative mouse from each group are shown as labeled. (C) The number of macroscopic tumor nodules in the lungs was counted in each mouse. The mean number of nodules per mouse was plotted for each group. Error bars represent 95% confidence intervals. (D) H&E stained slides of lungs from control and drug treated mice are shown at the same magnification. Arrows indicate metastatic lung tumors.

In the above orthotopic xenograft assay spontaneous metastasis of tumors in either control or treatment groups was not observed. To address the question of whether AZD1152 could block metastases of breast cancer as well as growth of primary tumors, a breast cancer xenograft model with lung metastasis potential was employed. MDA-MB-231 human breast cancer cells known to be highly metastatic were used in this experimental assay. Six to eight week old female athymic *nu/nu *mice were injected via tail vein with 2 × 10^6 ^MDA-MB-231 human breast cancer cells. Mice were randomized into 2 groups: control and AZD1152. Treatment with vehicle or AZD1152 (125 mg/kg/dose) began 2 days after intravenous injection of breast cancer cells. Drug or vehicle were administered IP on days 1 and 2 of a 7-day repeating cycle for 4 weeks only. Ten weeks after injection of cancer cells, mice were sacrificed, and the lungs were removed and weighed. The mean weight of lung tissue per mouse in the control group was significantly (P < 0.01, two-tailed t-test) higher than that in the AZD1152 group (Figure 5A). Further, the number of macroscopic tumor nodules was found to be significantly (P < 0.008, two-tailed t-test) higher in the lungs of control mice compared with AZD1152-treated mice (Figure 5C). Gross anatomic appearance of matched lung lobes from representative mice is shown in Figure 5B. H&E staining of lung tissues from control and AZD1152-treated mice showed reduction of tumor burden by AZD1152 at the microscopic level (Figure 5D). The data show that AZD1152 is effective in inhibiting the aggressive and highly metastatic phenotype of MDA-MB-231 human breast cancer cells *in vivo*.

### AZD1152 reduces Aurora B protein level by increasing polyubiquitination and degradation via the proteasome

**Figure 6:**
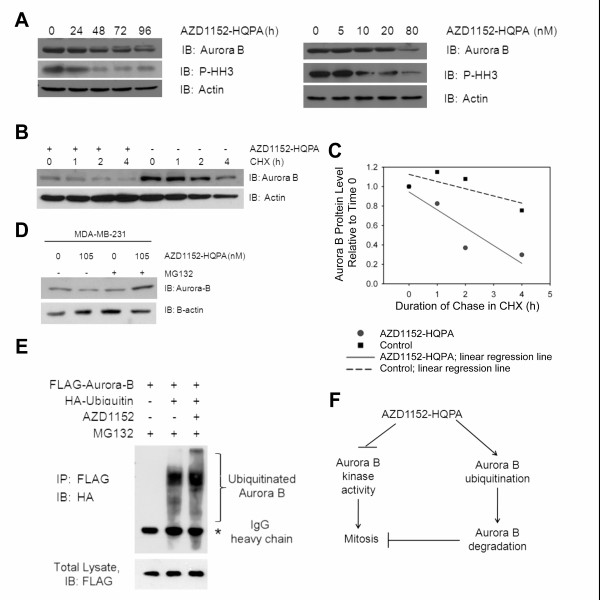
**AZD1152 reduces Aurora B protein level by increasing poly-ubiquitination and degradation via the proteasome**. (A) Western blots were performed for Aurora B, phospho-Histone H3 and actin levels. HER18 cells were treated with increasing duration of AZD1152 20 nM (left panel) and with increasing concentration of AZD1152 for 48 hours (right panel) as labeled. (B) HER18 cells were treated with or without 20 nM AZD1152 for 48 hours and then in the presence of cycloheximide for up to four hours. Immunoblots of Aurora B and actin are shown. (C) The integrated optical densities of the protein bands in B. The amount of Aurora B protein relative to 0 h of cycloheximide treatment was calculated for each group and corrected for gel loading differences based on actin. The relative rates of Aurora B turnover were estimated by the slope of the linear regression line for the control and AZD1152-HQPA-treated cells. (D) MDA-MB-231 cells show increased Aurora B protein levels when treated with the proteasome inhibitor MG132 and AZD1152-HQPA versus AZD1152-HQPA treatment alone. (E) AZD1152-HQPA causes increased poly-ubiquitinated Aurora B versus control. HER18 cells were transfected with the plasmids (Flag-Aurora B and HA-ubiquitin) and treated with the drugs (AZD1152-HQPA and the proteasome inhibitor MG132) as labeled above the top panel. Top panel: anti-HA immunoblot of anti-Flag-immunoprecipitates. Bottom panels: Anti-Flag immunoblot of whole cell lysates. (F) AZD1152-HQPA may disturb mitosis in at least two pathways: 1) through inhibition of Aurora B kinase activity, and 2) through increasing poly-ubiquitination and proteasomal degradation of Aurora B protein leading to decreased Aurora B function.

Examination of the impact of AZD1152-HQPA on Aurora B by immunoblotting also revealed that AZD1152-HQPA inhibits Aurora B kinase activity. This was reflected by a decrease in both phosphorylated Histone H3, and Aurora B protein level in a dose- and time-dependent manner (Figure 6A). The time courses showed that inhibition of Aurora B kinase activity preceded the decline in Aurora B protein level (Figure 6A). The unexpected finding of a decrease in Aurora B protein level was further investigated by examining the impact of AZD1152-HQPA on the protein turnover rate of Aurora B. HER18 cells were treated with or without 20 nM AZD1152-HQPA for 48 hours, then in the presence of cycloheximide (CHX) for up to four additional hours. After immunoblotting to measure Aurora B protein level (Figure 6B), the Aurora B protein band was quantified by densitometry. Relative protein level was calculated as the ratio of the integrated optical density of the Aurora B relative to time 0. The Aurora B protein turnover rates were determined as the slope of the linear regression line through each set of data points (Figure 6C). As indicated by the slope, AZD1152-HQPA increased the turnover rate of Aurora B. To test if the turnover of Aurora B was via the proteasome pathway, an experiment in which MDA-MB-231 cells were treated with or without AZD1152-HQPA in the presence of the proteasome inhibitor, MG132 (Figure 6D) was performed. The result shows that Aurora B protein levels were rescued by inhibition of the proteasome in the presence of AZD1152-HQPA.

Next, the ubiquitination of Aurora B in the presence of AZD1152-HQPA was investigated. HER18 cells were transfected with Flag-tagged Aurora B and HA-tagged ubiquitin. The transfected cells were incubated in the presence or absence of 20 nM AZD1152-HQPA. All samples were incubated with MG132 (10 μg/ml, 5 h) to inhibit the proteasome. Immunoprecipitation with anti-Flag antibody followed by immunoblotting with anti-HA antibody demonstrated the presence of polyubiquitinated Aurora B in cells transfected with both plasmids while no polyubiquitinated Aurora B and only the IgG heavy chain band was detected in cells transfected with Flag-Aurora B only (Figure 6E). Taken together, these results suggest that subsequent to inhibition of Aurora B kinase activity by AZD1152-HQPA, Aurora B protein turnover was increased by polyubiquitination and proteasomal degradation.

## Discussion

In this study, a knowledge gap regarding the use of an Aurora B kinase inhibitor in models of breast cancer was addressed. AZD1152-HQPA has the ability to inhibit a range of human breast cancer cell lines with IC_50_s in the range of 8-125 nM (Figure 1), which is similar to the range for leukemia [1] and colorectal cancer [7] cell lines. This concentration range is well below the concentrations where AZD1152-HQPA would inhibit Aurora A or other kinases [1]. Further, these six cell lines have various molecular profiles for HER2, ER, PR and p53. This is a potentially important finding in that this drug may provide an alternative method for treatment of breast cancer regardless of its molecular profile. One of the breast cancer cells lines assayed, MB-MDA 231 (HER2+ [16], ER-, PR-, p53 mutant), is considered to be a "triple negative" form of breast cancer. AZD1152-HQPA was also shown to be effective in this line which underscores the potential therapeutic value of this drug in a limited druggable-target form of breast cancer.

While investigating the cellular effects of AZD1152-HQPA it was found that it caused mitotic catastrophe resulting in aneuploidy, polyploidy and various other chromosomal abnormalities (Figure 2). Treatment with AZD1152-HQPA in breast cancer cells also led to apoptosis (Figure 3) as previously found in leukemia, multiple myeloma and colorectal cancer cells [1,7,10]. Additionally, it was found that colony forming potential in both anchorage dependent and anchorage-independent assays were reduced by this drug. These cell culture data prompted further investigation into the antineoplastic activity of AZD1152 in an *in vivo *model.

In an orthotopic xenograft model (Figure 4) and a lung metastasis model (Figure 5) of breast cancer, the *in vivo *antineoplastic effect of AZD1152 is demonstrated. The pharmacodynamic mechanism of inhibition of Aurora B kinase activity is confirmed *in vivo *by the decrease in phosphorylation of Histone H3 at serine 10 in xenografts as measured by immunoblotting with phospho-specific antibody. AZD1152 was well tolerated by the mice and no digestive distress or significant weight loss was observed. The *in vivo *antineoplastic effect demonstrated in these experiments warrants further investigation of this drug in clinical trials for breast cancer.

It has been discovered that in addition to inhibiting Aurora B kinase activity, AZD1152-HQPA also decreases Aurora B protein level in a dose- and time-dependent manner (Figure 6A). Mechanistic studies show that AZD1152-HQPA increases the turnover rate of Aurora B by increasing polyubiquitination and proteasomal degradation. The results uncover a previously unknown regulatory circuitry of AZD1152. Until now, no Aurora kinase inhibitor has been reported to affect the protein level of any Aurora kinase. These results also generate new hypotheses. Because Aurora B is autophosphorylated [25], it remains to be determined whether AZD1152 can cause inhibition of autophosphorylation, thereby enhancing the Aurora B degradation. Such a possibility will be another interesting layer of regulation to explore. These findings highlight the complexity of AZD1152's impact on post-translational regulation of Aurora B and provide the important insight that the inhibitory effect of AZD1152-HQPA may persist after wash-out of the drug from the target tissue. Theoretically, the inhibition of Aurora B should persist until the Aurora B protein level returns to normal. Therefore, the biological effects could be expected to last longer than the presence of the active inhibitor AZD1152-HQPA. This novel finding may aid the proper interpretation and use of pharmacokinetic and pharmacodynamic information about this drug to improve design of clinical dosing regimens.

## Conclusions

AZD1152-HQPA has antineoplastic activity against breast cancer cells in culture, and AZD1152 suppressed breast cancer growth in two mouse models. AZD1152-HQPA accelerated turnover of Aurora B protein by increasing poly-ubiquitination and proteasomal degradation. The finding that AZD1152-HQPA downregulates Aurora B protein level as well as inhibits Aurora B kinase activity (Figure 6F) will be important for the interpretation of pharmacokinetics and pharmacodynamics of AZD1152.

## Methods

### Cell lines

HER18 cells (stably expressing HER2) were described previously [19] and were cultured in DME/F12 media (Sigma, St. Louis, MO) supplemented with 10% (v/v) fetal bovine serum (Gemini, West Sacramento, CA). Human breast cancer cell lines MDA-MB-231, MDA-MB-435, MDA-MB-361, BT-474 and MDA-MB-468 were obtained from ATCC (Manassas, VA, USA). MDA-MB-231, MDA-MB-435 and MDA-MB-468 cells were cultured in Leibovitz's L15 media (Cellgro, Manassas, VA) with 10% (v/v) fetal bovine serum, 2 mM L-glutamine (Cellgro, Manassas, VA) and 1% (v/v) antibiotic-antimycotic solution (Invitrogen, Grand Island, NY). BT-474 and MDA-MB-361 were cultured in DME/F12 media (Sigma, St. Louis, MO) supplemented with 10% (v/v) fetal bovine serum (Gemini, West Sacramento, CA). All the cells were incubated at 37°C with 5% CO_2_.

### Compounds and antibodies

Antibodies used in Western blots include: anti-phospho-Histone H3 (p-HH3) (Upstate, Lake Placid, NY), anti-PARP (Cell Signaling, Danvers, MA), anti-Aurora B (Abcam, Cambridge, MA), anti-HA (Sigma) and anti-actin (Sigma). The protein synthesis inhibitor cycloheximide and proteasome inhibitor MG132 were purchased from Sigma Chemical Co. (St. Louis, MO).

AZD1152 prodrug and its metabolite, AZD1152-HQPA, were provided by Astra Zeneca Pharmaceuticals (Macclesfield, Cheshire, UK). AZD1152-HQPA was dissolved in 100% DMSO at 10 mM, and used at indicated durations and concentrations after dilution in tissue culture media. AZD1152 was dissolved in 0.3 M Tris pH 9.0 (Fisher, Pittsburgh, PA) and 0.2% DMSO (Fisher) at a maximum concentration of 20 mg/ml. The AZD1152 solution was made fresh for each round of injection into mice.

### MTT (3-(4,5-dimethylthiazol-2-yl)-2,5-diphenyltetrazolium bromide) assay

Cell lines were seeded at 5-20% confluence in 96-well microplates and allowed to attach for 24 hours. AZD1152-HQPA was serially diluted in appropriate complete media to the stated final concentrations. Plates were incubated for 2 to 5 days. Following incubation, 20 μl of 3-(4,5-dimethylthiazol-2-yl)-2,5-diphenyltetrazolium bromide (MTT) (Sigma) was added to each well. After incubation for 1 to 5 hours, the media were replaced by 200 μl 100% DMSO in each well. After mixing, the microplates were read with an MRX revolution plate spectrophotometer (Dynex, Technologies, Chantilly, VA) at 570 nm. Averages of at least 4 replicates were plotted and 50% inhibitory concentrations (IC_50_) were estimated based on sigmoidal curve fitting. For validation of MTT method in HER18 cells, cells from 6-cm-diameter dishes after similar treatment with AZD1152-HQPA were counted using a Z1 Coulter particle counter (Beckman Coulter, Fullerton, CA), and average counts from triplicate plates were plotted against concentration of AZD1152-HQPA.

### Evaluation of *in vivo *antineoplastic activity

Six- to eight-week old female athymic (nu/nu) mice (Experimental Radiation Oncology, MD Anderson Cancer Center, Houston, TX, USA) were housed in AAALAC approved barrier facilities on a 12-hour light/dark cycle, with food and water ad libitum. Mice were treated under approved protocols in compliance with the animal care and use guidelines of our institution, the USDA and the NIH. For the breast cancer orthotopic xenograft model, nude mice were injected with 8.5 × 10^6 ^HER18 human breast cancer cells in the mammary fat pad [26,27]. The nude mice were supplemented with weekly subcutaneous estradiol cypionate (Pfizer, NY, NY) injections (3 mg/kg/week) [20,21] starting 2 weeks prior to injection of tumor cells. Mice were randomized into 3 groups and treatment with AZD1152 prodrug began when tumors were measurable (approximately 50 mm^3^). Control mice received IP injections of vehicle (0.3 M Tris pH 9.0), low dose group received 62.5 mg/kg/day of AZD1152, and the high dose group received 125 mg/kg/day of AZD1152 on days 1 and 2 of a 7-day cycle for 3 cycles. Tumor measurements were taken every 2 to 3 days, and volumes were estimated with this formula: Length × Width^2^/2 [28]. Mice were sacrificed 24 days post tumor cell injection, and tumors were dissected. Tumor samples were snap frozen for Western blotting or fixed in formalin and paraffin embedded for histological analysis. Immunohistochemistry (IHC) was performed using the ABC kit (Vector Lab.) according to standard techniques. Photomicrographs were taken at 200× magnification with an Olympus IX70 microscope and Olympus DP controller imaging software (version. 3.2.1.276).

For the breast cancer lung metastasis model, nude mice were injected with 2 × 10^6 ^MDA-MB-231 cells via a tail vein. Cells were suspended in 200 μl complete media (DME/F12 with 10% fetal bovine serum). Mice were randomized into 2 groups (control and AZD1152). The AZD1152 group received injections of AZD1152 125 mg/kg/day IP on days 1 and 2 of a 7-day cycle for 4 cycles starting two days after tumor cell injection. Control mice received IP injections of vehicle (0.3 M Tris pH 9.0). Mice were sacrificed 10 weeks after tumor cell injection, and the lungs were weighed and examined for tumor nodules. Tumor samples were fixed in formalin and paraffin embedded for histological analysis.

### Flow cytometry

Breast cancer cells for cell cycle analysis were plated in 100-mm tissue culture dishes and allowed to attach for 24 hours. 100 nM AZD1152-HQPA or vehicle were applied to each plate for indicated times and cells harvested by trypsinization (Cellgro, Herndon, VA). Cells were collected and fixed in 70% ethanol for 1 hour followed by additional washing in PBS pH 7.40. Propidium iodide (PI) solution (50 μg/ml) with RNAseA (20 μg/ml) (Qiagen, Valencia, CA) was added to each sample and samples analyzed with a FACScalibur flow cytometer (Becton Dickinson, Franklin Lakes, NJ, USA). Cells analyzed for apoptosis were plated in 100-mm dishes and allowed to attach for 24 hours before addition of AZD1152-HQPA. Cells were washed with PBS and trypsinized at appropriate times for harvest. Cells were suspended in 0.5 ml binding buffer (Axxora, San Diego, CA) and 5 μl Annexin V-FITC (BD, San Jose, CA) for 15 minutes at room temperature in the dark. The cells were washed again, and PI solution with RNAseA was applied just before analysis.

### Colony forming assays

Soft agar colony forming assays were performed in triplicate plates with a base agar of 1× DME/F12 complete medium with 0.5% low melt agar (Fisher) and either DMSO for the control or 80 nM AZD1152-HQPA. Base agar was added to 6 cm Petri dishes and allowed to set. 5000 cells/plate added to 0.35% top agar with control medium or 80 nM AZD1152-HQPA. The plates were incubated at 37°C with 5% CO_2 _for 26 days. Colonies >100 cells were counted using a dissecting microscope, and results were analyzed using the Student's t-test.

Colony forming assays were performed in triplicate by trypsinizing breast cancer cells and plating them in 6-cm tissue culture dishes at 5000 cells/plate in complete DME/F12 medium with either DMSO or 40 nM AZD1152-HQPA. The plates were incubated for 12 days at 37°C with 5% CO_2 _for 12 days. Colonies were counted in 3 representative one cm^2 ^areas/plate and the number colonies per cm^2 ^between AZD1152-HQPA-treated cells and control cells were compared using Student's t-test.

### Fluorescence microscopy

Breast cancer cells were grown in chamber slides and treated with either DMSO or 20 nM AZD1152-HQPA for 48 hours. The chamber slides were rinsed 2× with cold PBS solution and fixed with 10% formaldehyde (Sigma). Cells were permeabilized with 0.2% Triton X-100 (Sigma), stained with DAPI (Invitrogen) and visualized with an Olympus IX81 fluorescent microscope.

## Competing interests

The authors declare that they have no competing interests.

## Authors' contributions

Experiments were performed by CG, FZ, JC, JY, GVT, and EW. CG, SCY and MHL conceived the experimental design. All authors approved the final manuscript.
